# Use of Preclinical Models to Improve Treatment of Retinoblastoma

**DOI:** 10.1371/journal.pmed.0020332

**Published:** 2005-10-25

**Authors:** Michael A Dyer, Carlos Rodriguez-Galindo, Matthew W Wilson

## Abstract

Dyer and colleagues examine the most promising preclinical models that recapitulate the molecular, genetic, and cellular features of retinoblastoma.

Retinoblastoma is a rare childhood cancer of the retina. Approximately one in 20,000 children are affected worldwide; only 250 to 300 new cases are reported in the United States each year. Enucleation (see Glossary) is usually the treatment of choice for children with unilateral disease. Treatment of children with bilateral retinoblastoma is more challenging, as eye and vision preservation become priorities. Historically, bilateral retinoblastoma was treated with a combination of laser therapy, cryotherapy, and radiotherapy. Today, patients with bilateral retinoblastoma first receive upfront chemotherapy to reduce the tumor burden, and then undergo aggressive focal therapies. This approach has increased the rate of eye salvage and decreases or delays the use of radiation therapy.

**Glossary**

**BAC-CGH:** Bacterial artificial chromosome-comparative genome hybridization. Large overlapping regions of the human and mouse genomes have been incorporated into bacterial artificial chromosomes. These DNA fragments are then spotted onto glass slides in an array, and can be used to hybridize two genomic samples labeled with different fluorochromes. If a particular genomic region is amplified or deleted in one sample, it will be revealed as an increase or decrease in the intensity of the fluorescence of that sample on the array. This approach is useful for identifying genomic changes in cancer cells as they progress from preneoplastic lesions to metastatic cancer.

**Chx10:** Most if not all retinal progenitor cells express the *Chx10* homeobox gene. Mutations in the *Chx10* gene cause microphthalmia in both humans and mice, indicating that the Chx10 protein is an important regulator of retinal progenitor cell proliferation. In addition, Chx10 is expressed in mature bipolar neurons and Müller glia of the mouse and human retina.

***Chx10-Cre;Rb^Lox/Lox^;p107^−/−^* genetic background:** Mice with conditional inactivation of Rb in retinal progenitor cells during retinal development. The *p107* gene is deleted in the entire mouse, so the retinal cells are lacking both *Rb* and *p107*. These mice develop retinoblastoma by several months of age.

**Cre transgenic lines:** A variety of transgenic mouse lines have been generated that express Cre recombinase under the control of tissue-specific promoters. For example, the *Chx10* promoter drives Cre expression in retinal progenitor cells, bipolar cells, and Müller glia.

**Cre:** A bacteriophage P1 protein that rapidly catalyzes site-specific recombination between two *LoxP* sites that are made up of a 34-bp DNA sequence.

**Drug-efflux transporters:** Some chemotherapeutic drugs are substrates for drug-efflux transporters that are expressed in the brain, and as a result, exhibit negligible uptake in the brain. If cancer cells express high levels of drug-efflux transporters, there may be an impact on how those cells respond to certain classes of chemotherapeutic agents.

**Epigenetic programs:** Genetic changes that result in alterations in gene expression are key features of many types of cancer. However, there are many examples of nongenetic changes in gene expression that contribute to cancer initiation or progression. These nongenetic or epigenetic changes can result from alterations in chromosomal structure or from condensation as a result of DNA methylation or histone organization.

**Enucleation:** Surgical removal of the eye from its orbit. Children with retinoblastoma who undergo enucleation often receive a prosthetic eye.

**Exon-specific knock-in mouse:** A genetically engineered mouse strain that encodes a single amino acid substitution in a single exon of the *Rb* gene. As a result of the exon-specific knock-in mutation, a normally spliced mRNA is produced that encodes a full-length Rb protein with a single amino acid substitution.

***Rb*** **null alleles:** An allele of *Rb* that is mutated and makes no functional protein. This is in contrast to some point-mutant alleles of *Rb* that retain partial function.

***Rb,p107*** **conditional knock-out mice:** The conditional allele of *Rb* called *Rb^Lox^*, contains two small DNA sequences called *LoxP* sites, which are recognition elements for the Cre recombinase. When Cre recombinase is introduced into the cell, it goes into the nucleus and deletes the DNA between the two *LoxP* sites, leading to a conditional knock-out tissue. There is not currently a *p107^Lox^* allele available so to inactivate both *Rb* and *p107* in the developing retina, *Rb^Lox/−^;p107^−/−^* mice were crossed to a transgenic mouse line expressing Cre recombinase in the developing retina. When *Rb* and *p107* are knocked out in the developing retina, retinoblastoma forms.

***Rb^−/−^*** **mice:** *Rb* knock-out mice carry a disrupted copy of the mouse ortholog of the human *RB1* tumor-suppressor gene. *Rb^−/−^* mice die in utero around day 13.5 of gestation due to hematopoietic and neural defects.

***Rb^Lox^***: A genetically engineered mouse line with two *LoxP* sites flanking one or more critical exons of the mouse *Rb* gene. When Cre recombinase is expressed in cells with a *Rb^Lox^* allele, the DNA between the *LoxP* sites is deleted, leading to a null allele of *Rb*.

***Rb^Lox/−^;p53^Lox/−^;p107^−/−^*** **mice:** These mice are similar to the *Rb^Lox/−^;p107^−/−^* mice described above, except that they also contain a conditional allele of the *p53* tumor suppressor. Conditional inactivation of *Rb*, *p53* and *p107* in the developing retina leads to aggressive invasive bilateral retinoblastoma in 100% of mice.

**SNP chips:** Single nucleotide polymorphisms are associated with particular genomic regions. Oligonucleotide arrays, or chips, have been developed that allow researchers to compare two DNA samples, and determine if there are amplifications or deletions.

***SV40*** **large T oncogene:** A viral oncogene from the Simian Virus 40 genome. Large T protein binds and inactivates the Rb and p53 tumor-suppressor proteins, and leads to deregulated cell proliferation.

**Two-photon live imaging:** Standard laser confocal imaging is not suitable for many in vivo, or live, imaging applications because the light cannot penetrate deep enough into the tissue, and the energy of the light that is required may kill the cells being studied. Two-photon imaging combines the energy of two photons that are half the energy and twice the wavelength on a single cell. The overall result is that images can be obtained much deeper in the tissue because of the longer wavelength, and the cellular toxicity is reduced because of the lower energy of the light being used.

Unfortunately, recent advances in targeted chemotherapy have not benefited patients with retinoblastoma because our knowledge is limited regarding the signaling pathways affected following the inactivation of the retinoblastoma gene *RB1*. Also, retinoblastoma clinical trials take years to complete because so few patients are available. Finally, pharmaceutical companies have little financial incentive to develop therapies for childhood cancers because pediatric patients represent only 1% of all patients with cancer. Therefore, preclinical models that recapitulate molecular, genetic, and cellular features of retinoblastoma are essential for identifying the most promising new therapies.

For decades, St. Jude Children's Research Hospital has brought together leading researchers and clinicians to tackle some of the most debilitating childhood diseases. This article is based on our recent symposium, “Retinoblastoma: From Bench to Bedside,” at which ophthalmologists, pediatric oncologists, and cancer researchers from around the world gathered to review the current status of retinoblastoma management, and to discuss the most effective ways to use the preclinical retinoblastoma models developed last year [1–5].

## Landmark Studies in Retinoblastoma Research

After studying retinoblastoma's pattern of inheritance susceptibility in families with a history of the disease, Alfred Knudson (Fox Chase Cancer Center) proposed a “two-hit” model to explain how a putative tumor suppressor could be inherited as a dominant trait that relied on inactivation of both alleles of the gene [6]. His colleagues studied the genetic lesions in children with heritable retinoblastoma and cloned the first human tumor-suppressor gene *RB1* [7], supporting Knudson's two-hit model. These important discoveries generated great enthusiasm for the possibility of improved therapies for retinoblastoma, as exemplified in the statement by David Abramson (Memorial Sloan-Kettering Cancer Center) in 1986: “Within fifteen years, at the outside, we'll be able to stop retinoblastoma before it begins. I'm so sure of that that I've already given the drug a name. I call it retino-revert. We'll be able to diagnose a child prenatally and start giving retino-revert to the mother to prevent retinoblastoma from growing as the fetus is developing” [8]. However, we are no closer today to identifying an effective targeted chemotherapy for retinoblastoma than we were when this statement was made. This slow progress can be attributed, in part, to the lack of preclinical models that recapitulate the disease.

The first mouse model of retinoblastoma was generated in 1990 by ectopically expressing the *SV40* large T oncogene in the developing retina [9]. The limitation of this model is that the SV40 T antigen may disrupt pathways that are not deregulated in human retinoblastoma. Thus, treatments that are effective in this model may have little clinical impact. In 1992, genetically engineered mouse strains carrying an inactivated *Rb1* gene were generated by three groups [10–12]. Unlike children with germline *RB1* mutations, *Rb1* mice did not develop retinoblastoma. In 1998, chimeric mouse studies demonstrated that *p107*, a gene related to *RB1*, was involved in preventing retinal tumor formation in mice [13]. Although these studies provided important clues about the resistance of mice to retinoblastoma, chimeric mice cannot be used as a preclinical model because of considerable interanimal variability and low efficiency of chimeric mouse generation.

## New Preclinical Models of Retinoblastoma

Childhood central nervous system (CNS) tumors arise during fetal or early childhood development. A major hurdle in the development of preclinical retinoblastoma models has been our limited knowledge about normal neural development. In other words, we have greater understanding of the molecular genetics of retinoblastoma than of the normal developmental neurobiology of the retina. Over the past ten years, Connie Cepko (Harvard University) and many others have advanced the field of retinal development, so that we now have a context in which to understand the cancer's biology [14,15].

Retinoblastoma research marked 2004 as an important year. Researchers combined developmental neurobiology with cancer biology to elucidate retinoblastoma formation, and those findings are now being moved directly into the clinic [1,5,16]. Advances were made in three key areas. First, the role of Rb protein in normal retinal development was elucidated. The *Rb* gene regulates two distinct processes in the developing mouse retina: rod photoreceptor development and progenitor cell proliferation (Schweers B, Donovan SL, Gray J, Zhang J, Martins R, et al., unpublished data) [5,17]. In *Rb^−/−^* mice, p107 partially compensates for Rb in the latter process but not the former, indicating that the two genes have both shared roles and unique roles (Schweers B, Donovan SL, Gray J, Zhang J, Martins R, et al., unpublished data) [5]. This work is an extension of Julien Sage's (Stanford University) work on p107 compensation after *Rb* inactivation in mouse embryonic fibroblasts [18].

**Figure 1:**
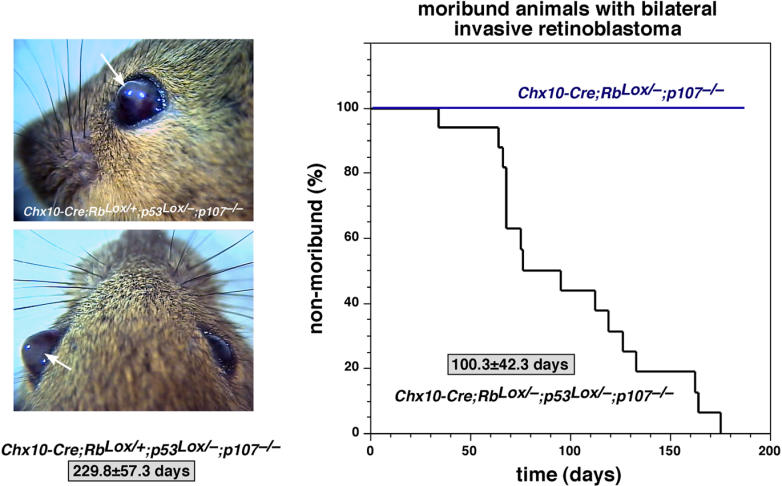
p53 Prevents Invasive, Aggressive Retinoblastoma in Mice *Rb* was conditionally inactivated in retinal progenitor cells by using the *Chx10-Cre* transgenic mouse line and the *Rb^Lox^* allele. On a *p107*-deficient genetic background, these mice develop retinoblastoma with a penetrance of approximately 60%. However, the disease rarely progresses to invasive retinoblastoma with ocular hypertrophy, and the mice rarely become moribund. In contrast, mice lacking both copies of *p53*, *Rb*, and *p107 (Chx10-Cre;Rb^Lox/−^;p53^Lox/−^;p107^−/−^)* in their retinal progenitor cells develop aggressive, invasive retinoblastoma. More importantly, penetrance is 100% as bilateral retinoblastoma with a short latency (100.3 ± 42.3 days), which is ideal for testing new chemotherapeutic drugs. Interestingly, mice with one copy of wild-type *Rb* also develop invasive, aggressive retinoblastoma, but with a longer latency than do *Chx10-Cre;Rb^Lox/−^;p53^Lox/−^;p107^−/−^* mice. Preliminary studies indicated that these tumors have lost heterozygosity at the *Rb* locus; thus, *Chx10-Cre;Rb^Lox/−^;p53^Lox/−^;p107^−/−^* mice are the only mouse model of retinoblastoma that recapitulates this feature of human retinoblastoma.

The second advance was the generation of a mouse carrying targeted deletions of *Rb* and *p107* in the developing retina [1,19]. By several weeks of age, the eyes of *Rb,p107* conditional knock-out mice filled with immature, proliferating retinal cells, which gave rise to retinoblastoma [1]. Shortly after these studies were published, two other groups published similar findings using Cre transgenic lines with broader expression patterns in the retina *(Pax6-Cre)* [3] and in the developing CNS *(Nestin-Cre)* [2]. Unfortunately, neither *Pax6-Cre* nor *Nestin-Cre* mice can be used as preclinical models because of low penetrance, late onset, and nonautonomous effects (cell nonautonomous effects are phenotypic changes that occur in the tissue or cell being studied as a result of a change in the environment, rather than loss of the gene of interest in the tissue or cell itself). The importance of the p53 pathway in retinoblastoma was shown in mice with retinas lacking *Rb*, *p107*, and *p53* [1]. These mice developed bilateral retinoblastoma with 100% penetrance and a short latency; thus, they are ideal for testing new chemotherapeutic drugs (Figure 1). Also, their mosaic pattern of Cre expression in *Chx10-Cre* mice is a more appropriate model of human retinoblastoma than *Pax6-Cre* or *Nestin-Cre* (discussed in [1,20]).

The third advance in retinoblastoma research followed the generation and characterization of two other preclinical models. This work has not been published but was presented at the symposium “Retinoblastoma: From Bench to Bedside”; the Dyer laboratory and St. Jude have made these models directly available to the retinoblastoma research community. Studies on these animal models were combined with comprehensive cell culture, molecular biology, and pharmacokinetic studies to investigate drug efficacy for the treatment of retinoblastoma [16]. Analyses of five drugs and their combinations revealed that topotecan combined with carboplatin is a feasible alternative to the current triple-drug therapy (i.e., etoposide, vincristine, and carboplatin). These preclinical studies may provide useful information to complement the ongoing RET-5 clinical protocol at St. Jude (C.R. and M.W.W.) focused on the use of topotecan-combination chemotherapy.

## Strengths and Limitations of Current Preclinical Models of Retinoblastoma

**Figure 2:**
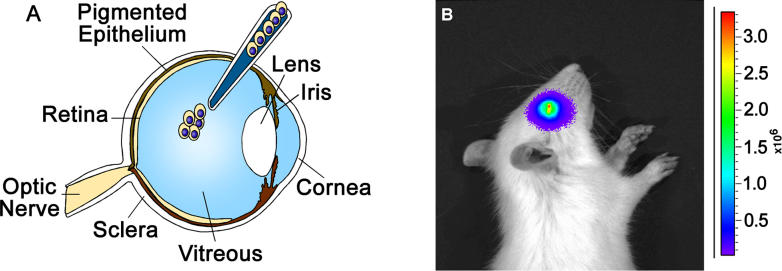
A Developmentally Appropriate Orthotopic Retinoblastoma Xenograft Model (A) Injection of 1,000 cultured human retinoblastoma cells into the vitreal cavity of newborn rat eyes leads to retinoblastoma by two weeks of age. The rats do not require immunosuppression because they are immunonaive for the first 24 hours after birth. This is an ideal model for testing new drugs and for studying the retinoblastoma cancer stem cell properties. (B) Human retinoblastoma cells have been genetically engineered to express the luciferase reporter gene. A two-week-old rat that received an intravitreal injection of Y79-LUC cells at birth is shown. Y79-LUC cells are retinoblastoma cells that express the firefly luciferase gene under the control of a constitutive promoter (see [16]). At two weeks, the rat received an intraperitoneal injection of the luciferase substrate (luciferin). The fluorescence is directly proportional to the tumor volume and the number of cells in the tumor. By using the Xenogen imaging system, we can follow individual tumors as they grow and respond to chemotherapy.

An orthotopic xenograft model has been developed for retinoblastoma, which relies on injecting human retinoblastoma cells into the eyes of newborn rats (Figure 2) [16]. At the symposium, there was great enthusiasm for the orthotopic xenograft model developed at St. Jude [16], and with new cell lines or primary tumors directly injected into the eyes of newborn rats, this xenograft model could be improved further (Figure 2). Enthusiasm for the clonal focal model involving the *E1A 13S* retrovirus in p53-deficient mice [16] was dampened by the use of an oncogene (Schweers B, Donovan SL, Gray J, Zhang J, Martins R, et al., unpublished data) [4,21] that could disrupt pathways that are not inactivated in human tumors. Briefly, a replication-incompetent retrovirus expressing the *E1A 13S* oncogene is injected into the subretinal space of *p53*-deficient newborn mice. Within a few weeks, these mice develop focal clonal retinoblastoma (Schweers B, Donovan SL, Gray J, Zhang J, Martins R, et al., unpublished data) [4,16,21]. The approach using Cre retrovirus injected in the eyes of newborn *Rb^Lox/−^;p53^Lox/−^;p107^−/−^* mice (discussed in [4]) was considered at the symposium to be superior to that using the *E1A* oncogene; however, the Cre-retrovirus model relies on *p53* inactivation, which is not mutated in human tumors. One suggestion at the symposium was to identify the genetic lesion that disrupts the p53 pathway in primary human tumors, and then genetically engineer mice with the same mutation.

Two additional concerns about the current models were discussed. One is the use of *Rb* null alleles. Children with retinoblastoma often have a mutated *RB1* allele and a null allele. David Goodrich (Roswell Park Cancer Institute) and colleagues have created an exon-specific knock-in mouse with a single amino acid change found in human retinoblastoma (Sun H, Yanjie C, Zhang X, Schweers B, Dyer MA, et al., unpublished data). The knock-in allele was crossed with the *Chx10-Cre;Rb^Lox/Lox^;p107^−/−^* and *Chx10-Cre;Rb^Lox/Lox^;p107^–/–^;p53^Lox/Lox^* genetic backgrounds. The single amino acid change at residue 654 is a low-penetrance allele of *RB1* in humans; it will be interesting to determine if mice have a similar phenotype. The second concern is that models with two germline mutations do not recapitulate human retinoblastoma. In sporadic retinoblastoma, acute mutations are believed to occur at both *RB1* loci; in heritable retinoblastoma, one allele has a germline disruption and the other undergoes acute inactivation. This concern is strengthened by Sage's finding that the timing of *Rb1* inactivation (chronic versus acute) influences *p107* compensation [18]. In these experiments, *p107* compensation refers to the upregulation of *p107* gene and protein expression when Rb expression is lost. By combining the knock-in point-mutant *Rb* allele described above with Cre-expressing retroviruses, we can more accurately recapitulate human retinoblastoma.

Another topic extensively discussed was the role of the p53 pathway in retinoblastoma. Clearly, p53 inactivation accelerates retinoblastoma in mice and leads to more aggressive, invasive tumors (see Figure 1) [1]. However, whether the p53 pathway is inactivated in human retinoblastoma is unknown. Several possible mechanisms were presented for the inactivation of the p53 pathway when the *p53* gene is intact, as in retinoblastoma. It is particularly important that *p53* is intact because it may provide targets for chemotherapy. Comprehensive analysis of this pathway in retinoblastoma should be a key focus of future research.

## Using Preclinical Data in Support of Clinical Trials

One of the major obstacles to testing new chemotherapeutic combinations over the past 10–12 years has been the lack of a preclinical model that faithfully recapitulates the human disease. We are now able to merge our understanding and research of the intraocular physiology and drug kinetics with the unique biology of retinoblastoma (both in its initiation and in its temporal progression) in the same model. Researchers are now given the opportunity to answer some of the critical questions that the new era of conservative management has posed. The preclinical models have been successfully used to test new drugs and combinations [1,16]. However, more is still expected from these models, as many relevant questions have been left unanswered by clinical trials. More information regarding new agents, correct combinations and schedules, and route of administration (systemic or periocular) is needed.

It is clear that one of the major hurdles for future progress is suboptimal communication between the different clinical and translational researchers. This hurdle is compounded by the fact that there is still substantial disagreement among treating physicians about the objectives of the treatment and the means (treatment regimens) to achieve them. A simple question such as what constitutes the standard of care for a child with bilateral retinoblastoma is not easy to answer. For example, carboplatin as a single-drug regimen can provide ocular salvage that is as good as more aggressive regimens that also incorporate etoposide and vincristine, but the efficacy of both regimens depends on the stage of the intraocular disease and the appropriate use of aggressive focal treatments by an expert team.

It is not surprising then that single-drug carboplatin therapy is used by some institutions, while others use the two-drug carboplatin–vincristine combination, the more standard three drug combination that incorporates etoposide, or a modification of the latter that adds cyclosporine to inhibit drug-efflux transporters that might be involved in drug resistance. Because of the limited number of patients with retinoblastoma that are eligible for chemotherapy clinical trials, a proper comparison of those treatment regimens may not be possible.

While these questions are being proposed, new ideas are being brought into the equation. For example, the unique anatomy and physiology of the eye creates an environment where cancer remains well contained until advanced stages. In this context, increasing the intraocular concentration of antineoplastic agents through modulation of their intraocular pharmacokinetics, and developing new methods of direct intraocular delivery of drugs will become priorities. Even more challenging are efforts to design drugs that treat (or prevent the development of) retinoblastoma in utero, or to generate different mouse models that recapitulate the different human *RB1* mutations. Gene therapy is also an interesting option, but this therapy has its own unique challenges, such as spread of the virus and secondary toxicity to the surrounding normal retina. How to incorporate these innovations into current regimens will become a major challenge in the future. Here again, the information gathered in the preclinical models will be pivotal to the successful translation of these innovations into the frontline treatments.

Input from clinicians and pharmacologists is essential for designing experiments that will be clinically relevant. Pharmocokinetics, side effects, dose, and schedule are essential considerations when performing preclinical studies for retinoblastoma. For this reason, a retinoblastoma working group that includes pharmacologists, pathologists, neurobiologists, ophthalmologists, and pediatric oncologists, such as the one at St. Jude is essential for effectively moving new treatments tested in the lab into clinical trials. Just as the 20th century saw improved outcomes and decreased morbidity for retinoblastoma, so may the 21st century, with targeted chemotherapy and new methods of drug delivery into the eye. In this new era of translational research, experts from several different fields must work together in the delicate process of identifying and resolving the challenges posed by this unique malignancy. Just as retinoblastoma has been at the forefront of many of the advances in cancer biology over the past several decades, retinoblastoma researchers are in a unique position to serve as the example for translational research (see [22]).

## Retinoblastoma Provides a Unique Window into Tumor Growth and Death

**Figure 3:**
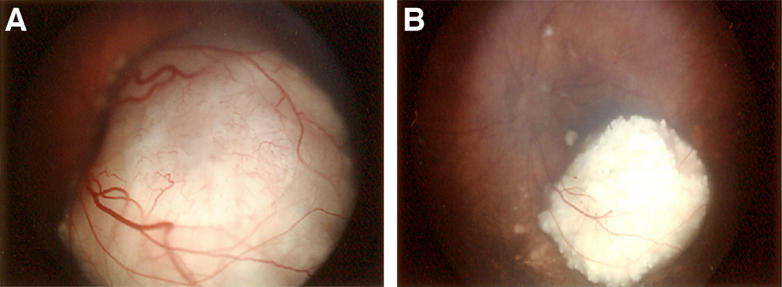
The Eye Is a Transparent System That Permits Direct Visualization of Retinoblastoma (A) Untreated retinoblastoma involving the macula of the left eye. The translucent cornea and lens allow detailed visualization. The tumor is an amelanotic, partially calcified mass that has broken through the overlying retina. Dilated arterioles infiltrate the tumor. (B) The same tumor after being treated with chemotherapy. The mass has completely calcified, and the caliber of the overlying retinal vessels has diminished. A focal area of atrophied retinal pigmented epithelium surrounds the lesion.

Advances in retinoblastoma treatment are, in part, due to the unique environment of the eye––a transparent system that permits direct visualization of the disease (Figure 3). A clear cornea, lens, and vitreous are inherent to successful management, e.g., transparency is imperative for precise delivery of focal therapies. Imaging modalities (i.e., ultrasonography, computed tomography, and magnetic resonance imaging) can be used to confirm the diagnosis but not to assess the response to treatment. Only with indirect ophthalmoscopy and scleral depression can the entire retina be examined. Also, tumor recurrences smaller than 1 mm and early vitreous seeding are easily appreciated by the trained observer; this resolution is not attainable by other imaging techniques.

Future treatment regimens will continue to rely on the unique properties of the eye; however, they will exploit the eye's permeability rather than transparency. A substantial body of work has already begun to document the efficacy of periocular chemotherapy [23,24]. The volume, concentration, and toxicity of agents are limiting current endeavors; however, investigators are exploring both new drugs and new vehicles of delivery to achieve efficacious ocular concentrations.

## Future Directions: Targeted Chemotherapy

To more efficiently target chemotherapy to retinoblastoma and move away from broad-spectrum agents, we need to answer three fundamental questions: what is the disease's cell-of-origin, how does it expand, and what genetic events occur after *RB1* inactivation?

Typically, the cancer cell of origin is inferred from the expression of cell type–specific markers or other morphologic features. In CNS cancers, marker expression is difficult to interpret because the cell types are complex. Neurobiologists define normal cell populations by marker expression, dendritic and axonal morphology, and location within the CNS. These features are often lost or disrupted in CNS tumor cells; therefore, it is difficult to determine their cell of origin. The use of Cre-expressing retroviruses and two-photon live imaging will allow us to follow individual tumor clones as they expand beyond single-infected retinal progenitor cells, and help to identify the retinoblastoma cell of origin in mouse models (discussed in [4]). Identifying the retinoblastoma cell of origin is essential because different cells may rely on entirely different pathways to drive proliferation and tumor expansion. Verification that the cell of origin in mouse retinoblastoma is the same as that in human retinoblastoma is essential for successful use of the model in screening new drugs targeted to deregulated pathways in that cell.

Second, we need to determine if retinoblastoma expands by a cancer stem cell mechanism [25,26] or by expansion of all cells in the tumor. A tumor can expand from a small population of cancer stem cells, even if it did not arise from a normal stem cell. This is of particular importance in retinoblastoma because no stem cells are present where tumors arise in the neural retina [27,28]. The xenograft model (see Figure 2) is ideally suited for determining if retinoblastoma expands by a stem cell mechanism. Human and mouse tumors could be fractionated, serially transplanted into the developing eye, and followed to determine which cells reconstitute the entire tumor. Then, by efficiently targeting those cells, we could halt tumor progression without dramatically altering tumor volume. If all cells expand, then targeted chemotherapy will need to focus on halting the expansion of all cells in the tumor.

Third, we must identify the secondary genetic events that follow *RB1* inactivation. Processes such as escape from apoptosis (programmed cell death), deregulated growth, and telomere maintenance (capping of the ends of chromosomes) occur in most (if not all) cancers [29], but not all retinoblastomas achieve these changes through common genetic mechanisms. Indeed, the retinoblastoma cell of origin may be a heterogeneous population with diverse epigenetic programs, which may hinder the identification of common pathways for targeting chemotherapy. Molecular genetic analysis of primary human retinoblastomas and mouse retinoblastomas by gene expression microarrays, BAC-CGH, and single-nucleotide-polymorphism (SNP) chips provides a good starting point. Information on deregulated pathways in retinoblastoma can then be exploited to target chemotherapy and, ultimately, to halt tumor progression without the side effects associated with broad-spectrum chemotherapy.

## Conclusion

Developing new treatments for a complex disease such as retinoblastoma requires expertise in pediatric oncology, ophthalmology, pharmacology, and developmental neurobiology. The open forum at the “Retinoblastoma: From Bench to Bedside” symposium provided a unique opportunity for experts in each of these areas to discuss the best way to take advantage of recently developed preclinical models of retinoblastoma in order to achieve the common goal of saving vision in children with this devastating cancer.

While there is disagreement about the best treatment, there was a broad consensus about the importance of preclinical models to test new therapies prior to clinical trials. By combining preclinical models with analysis of primary human tumors, we hope to move away from broad-spectrum chemotherapy and its associated side effects, and more effectively target the pathways that are deregulated in retinoblastoma.

For further information, please see http://www.stjude.org/retinoblastoma, http://www.stjude.org/dyer, and http://www.cure4kids.net.

## Abbreviations

CNS: central nervous system
St. Jude: St. Jude Children's Research Hospital

## References

[pmed-0020332-b1] Zhang J, Schweers B, Dyer MA (2004). The first knockout mouse model of retinoblastoma. Cell Cycle.

[pmed-0020332-b2] MacPherson D, Sage J, Kim T, Ho D, McLaughlin ME (2004). Cell type-specific effects of Rb deletion in the murine retina. Genes Dev.

[pmed-0020332-b3] Chen D, Levine-bar I, Vanderluit JL, Slack RS, Agochiya M (2004). Cell-specific effects of RB or RB/p107 loss on retinal development implicate an intrinsically death-resistant cell-of-origin in retinoblastoma. Cancer Cell.

[pmed-0020332-b4] Dyer MA, Bremner R (2005). The search for the retinoblastoma cell of origin. Nat Rev Cancer.

[pmed-0020332-b5] Zhang J, Gray J, Wu L, Leone G, Rowan S (2004). Rb regulates proliferation and rod photoreceptor development in the mouse retina. Nat Genet.

[pmed-0020332-b6] Knudson A (1971). Mutation and cancer: Statistical study of retinoblastoma. Proc Natl Acad Sci U S A.

[pmed-0020332-b7] Friend SH, Bernards R, Rogelj S, Weinberg RA, Rapaport JM (1986). A human DNA segment with properties of the gene that predisposes to retinoblastoma and osteosarcoma. Nature.

[pmed-0020332-b8] Angier N (1988). Natural obsessions: The search for the oncogene.

[pmed-0020332-b9] O'Brien JM, Marcus DM, Bernards R, Carpenter JL, Windle JJ (1990). A transgenic mouse model for trilateral retinoblastoma. Arch Ophthalmol.

[pmed-0020332-b10] Jacks T, Fazeli A, Schmitt EM, Bronson RT, Goodell MA (1992). Effects of an Rb mutation in the mouse. Nature.

[pmed-0020332-b11] Lees E, Faha B, Dulic V, Reed SI, Harlow E (1992). Cyclin E/cdk2 and cyclin A/cdk2 kinases associate with p107 and E2F in a temporally distinct manner. Genes Dev.

[pmed-0020332-b12] Clarke AR, Maandag ER, van Roon M, van der Lugt NM, van der Valk M (1992). Requirement for a functional Rb-1 gene in murine development. Nature.

[pmed-0020332-b13] Robanus-Maandag E, Dekker M, van der Valk M, Carrozza ML, Jeanny JC (1998). p107 is a suppressor of retinoblastoma development in pRb-deficient mice. Genes Dev.

[pmed-0020332-b14] Livesey FJ, Cepko CL (2001). Vertebrate neural cell-fate determination: Lessons from the retina. Nat Rev Neurosci.

[pmed-0020332-b15] Dyer MA, Cepko CL (2001). Regulating proliferation during retinal development. Nat Rev Neurosci.

[pmed-0020332-b16] Laurie NA, Gray JK, Zhang J, Leggas M, Relling M (2005). Topotecan combination chemotherapy in two new rodent models of retinoblastoma. Clin Cancer Res.

[pmed-0020332-b17] Donovan SL, Dyer MA (2004). Developmental defects in Rb-deficient retinae. Vision Res.

[pmed-0020332-b18] Sage J, Miller AL, Perez-Mancera PA, Wysocki JM, Jacks T (2003). Acute mutation of retinoblastoma gene function is sufficient for cell cycle re-entry. Nature.

[pmed-0020332-b19] Rowan S, Cepko CL (2004). Genetic analysis of the homeodomain transcription factor Chx10 in the retina using a novel multifunctional BAC transgenic mouse reporter. Dev Biol.

[pmed-0020332-b20] Schweers BA, Dyer MA (2005). New genetic tools to study retinal development in vivo. Vis Neurosci.

[pmed-0020332-b21] Dyer MA (2004). Mouse models of childhood cancer of the nervous system. J Clin Pathol.

[pmed-0020332-b22] Knudson A (2005). Retinoblastoma: Teacher of cancer biology and medicine. PLoS Med.

[pmed-0020332-b23] Hayden BH, Murray TG, Scott IU, Cicciarelli N, Hernandez E (2000). Subconjunctival carboplatin in retinoblastoma: Impact of tumor burden and dose schedule. Arch Ophthalmol.

[pmed-0020332-b24] Van Quill KR, Dioguardi PK, Tong CT, Gilbert JA, Aaberg TM (2005). Subconjunctival carboplatin in fibrin sealant in the treatment of transgenic murine retinoblastoma. Ophthalmology.

[pmed-0020332-b25] Lapidot T, Sirard C, Vormoor J, Murdoch B, Hoang T (1994). A cell initiating human acute myeloid leukaemia after transplantation into SCID mice. Nature.

[pmed-0020332-b26] Pardal R, Clarke MF, Morrison SJ (2003). Applying the principles of stem-cell biology to cancer. Nat Rev Cancer.

[pmed-0020332-b27] Tropepe V, Coles BL, Chiasson BJ, Horsford DJ, Elia AJ (2000). Retinal stem cells in the adult mammalian eye. Science.

[pmed-0020332-b28] Coles BL, Angenieux B, Inoue T, Del Rio-Tsonis K, Spence JR (2004). Facile isolation and the characterization of human retinal stem cells. Proc Natl Acad Sci U S A.

[pmed-0020332-b29] Vogelstein B, Kinzler KW (2004). Cancer genes and the pathways they control. Nat Med.

